# Susceptibility to smoking and determinants among medical students: A representative nationwide study in China

**DOI:** 10.18332/tid/106188

**Published:** 2019-04-24

**Authors:** Sihui Peng, Lingwei Yu, Tingzhong Yang, Dan Wu, Joan L. Bottorff, Ross Barnett, Shuhan Jiang

**Affiliations:** 1 Center for Tobacco Control Research, Zhejiang University School of Medicine, Hangzhou, China; 2 Children’s Hospital/Center for Tobacco Control Research, Zhejiang University School of Medicine, Hangzhou, China; 3 Department of Psychology, Guangdong Medical University, Dongguan, China; 4 School of Nursing, University of British Columbia, Kelowna, Canada; 5 Department of Geography, University of Canterbury, Christchurch, New Zealand; 6 School of Humanities and Management, Zhejiang Chinese Medical University, Hangzhou, China

**Keywords:** susceptibility to smoking, tobacco control, college students, China

## Abstract

**INTRODUCTION:**

The rationale behind why the majority of medical students are non-smokers, but some initiate smoking after becoming physicians is not fully understood in China. Exploring factors that may increase susceptibility to smoking initiation among medical students is an essential first step in assessing preventative actions.

**METHODS:**

Participants were 11954 students, who were identified through a multistage survey sampling process that included 50 universities in China. Subsequent analysis focused on 8916 non-smokers among medical students. Both unadjusted and adjusted logistic methods were considered in the data analyses.

**RESULTS:**

The prevalence of susceptibility to smoking was 23.0%. Multivariate logistic regression analyses found that exposure to secondhand smoke (SHS) in domestic places (OR= 1.63) and in public places (OR=1.78), cigarette advertising (OR=1.91) and promotional activities on campus (OR=1.90) were positively associated with susceptibility to smoking. In contrast, positive attitudes toward tobacco control on the part of health professionals, HPs, (OR=0.52) were negatively associated with susceptibility to smoking. Those who received information about the dangers of smoking (OR=0.75) and did not agree that light cigarettes are less harmful to health (OR=0.79) were less susceptible to smoke. Caring about exposure to secondhand smoke (OR=0.68 care, and OR=0.33 very) and advising family members to stop smoking (OR=0.81) were negatively associated with susceptibility to smoking.

**CONCLUSIONS:**

These findings underscore the importance of tobacco control training and establishing smoke-free campuses for reducing susceptibility to smoking among medical students.

## INTRODUCTION

Smoking is a behavioral change that may occur gradually through several stages of preparation, initiation, experimentation, regular smoking, and addiction^1,2^. Susceptible non-smokers are defined as people who are currently not smoking, but who are predisposed or motivated to start smoking in the future. Thus, susceptibility to smoking is defined as the absence of a firm decision not to smoke. Likewise, non-susceptibility is defined as the existence of a determined decision not to smoke^3,4^. Many studies have found that an early susceptibility to smoking is a strong predictor of smoking initiation^3,5,6^ but there has been some debate regarding the extent to which susceptibility is a direct predictor of smoking initiation^7^. Researchers have also found that susceptibility to smoking increases participation in tobacco industry promotion campaigns^8,9^, as well as decreased responsiveness and compliance with tobacco prevention programs^2,10^.

While such studies have proved valuable in conceptualizing the factors that lead to smoking initiation among youth, there are a number of critiques that can be made. First, while there are many studies that have explored adolescent susceptibility to smoking, these have mostly been confined to high-income countries^8^, and few studies have examined susceptibility in low- and middle-income countries^1,11^. Second, most of the studies have examined adolescents, and only a few have focused upon young adults and college students^12,13^. To the best of our knowledge, none has focused on medical students. Third, most research has focused on individual-level factors, yet ecological models emphasizing behavioural events are influenced by both individual and environmental variables^14^. Thus, it is important also to consider the latter and the way environmental factors may influence susceptibility^15^. For example, a number of studies have found that susceptibility to smoking reflects the intensity of tobacco industry promotion campaigns^8,9,16^, while other studies have shown clear associations between weak smoke-free policies, exposure to secondhand smoke (SHS) and susceptibility to smoking^2,12^.

Smoking prevalence remains high in China and medical college students, as future health professionals (HPs), are important agents for tobacco control. Health professionals play a critical role in improving services, advocating policies and serving as role models for social change. However, the smoking prevalence among physicians is much higher (23%) in China, especially among males^16^ (41%), compared to many other countries, particularly developed countries (about 6%)^17,18^. Compared with the smoking prevalence (33%) of the general population, the physician smoking ratio is 0.70 (23/33) in China, while this ratio is only 0.30 (6/20) in the western world^17^. This is a matter of concern because physician smoking not only affects their own health but also undermines the effective delivery of smoking cessation counselling to patients^18-20^. Jiang et al.^17^ in their study of 3552 hospital-based physicians in six Chinese cities reported that only 30% HPs reported good implementation of smoke-free workplace policies and 37% (of current smokers) had smoked in front of their patients^16^. Such trends are reminiscent of HPs in western countries such as the United States where cigarette smoking prevalence among physicians in 1974 (18.8%) was similar to that of China’s HPs 30 years later^21^.

Some medical students who are non-smokers begin to smoke after becoming physicians but the reasons for this are not fully understood. Some studies have suggested that social factors are important, such as the lack of anti-smoking norms, work stress, or physicians accepting cigarettes as gifts from patients^16,22^. Some physicians begin smoking as their attitudes, and practices of smoking start to reflect their experiences, and changes in their health behavior gradually begin to occur^14^. Exploring factors that may increase susceptibility to smoking initiation among medical students therefore is an important step in developing prevention strategies to reduce smoking rates among future physicians.

Most medical students in China eventually work in the healthcare industry including public hospitals (majority of Chinese hospitals), private clinics, and other healthcare units after graduation, as undergraduate, Masters or Doctoral students^23^, and nearly all of them work as physicians in these institutes^24^. Susceptibility to smoking as medical students may be a risk factor for the physicians’ actual smoking behaviours^3,5,6^, so that examining the susceptibility to smoking of medical students is important to understanding physicians’ smoking behaviour.

This study, therefore, focuses on examining susceptibility to smoking and its determinants among medical students by assessing three questions: 1) What is the prevalence of susceptibility to smoking among medical students who have never smoked?; 2) What environmental factors influence medical students’ susceptibility to smoke?; and 3) What kind of individual tobacco control attitudes, and university curriculum practices are associated with susceptibility to smoking?

## METHODS

### Study area and participants

This study reports individual data from the Global Health Professions Student Survey (GHPSS) on Tobacco Control in China GHPSS^11^. This study employed a multi-stage sampling design. In Stage 1, 60 potential universities with medical programs were identified across regions in China. Fifty of the 60 universities actually completed the survey process. These universities were distributed throughout China (Northeast, Northwest, North, Southeast, Southwest and South). Twenty-two were medical universities offering mainly medical professional programs, and 28 were comprehensive universities offering medical professional and non-medical professional programs. Stage 2 of the sampling strategy involved the selection of levels within each university. All levels that had medical professional courses were selected in each university. In Stage 3, one-third of those classes were randomly selected from each level. In Stage 4, all students in these selected classes were surveyed. A detailed description of the study methods can be found in Yang et al.^22^.

### Data collection

Data were collected using a self-report method. Once participants were identified and agreed to participate in the study, a structured self-administered questionnaire was used. The questionnaire was administered during regular class sessions and took approximately 30 minutes to complete. All responses were anonymous. A common research protocol was used across all 50 universities to ensure homogeneity of questionnaire administration and data collection techniques. A detailed description can be found in Yang et al.^22^. For the purpose of this study, only medical students who were never smokers were included in the analysis. Never-smoking medical students were identified as those participants who responded ‘no’ to smoking at present or in the past. This study was approved by the Ethics Committee at the Medical Center, Zhejiang University, and verbal consent was obtained from all participants prior to data collection.

### Measures

#### Dependent variable

The dependent variable is susceptibility to smoking among never-smoking medical students and was obtained referencing three questions similar to those developed by Pierce et al.^3^. These considered social influences on smoking initiation in China^14^. The questions were: 1) ‘Do you think that you will try a cigarette soon?’; 2) ‘If some of your best friends smoke in the future, will you smoke?’; and 3) ‘If social contact and work need you to smoke in the future, will you smoke?’. The following four-point ordinal scale was used: 1) definitely not, 2) probably not, 3) probably yes, and 4) definitely yes. Never-smoking medical students were identified as those participants who responded ‘no’ to smoking at present or in the past. Using previously described methods^8^, those who responded ‘definitely not’ to all three questions were considered as non-susceptible and recoded as ‘0’, and all other respondents were considered susceptible to smoking and recoded as ‘1’.

Cronbach’s coefficient among three questions to measure susceptible to smoking was 0.7527. The first question in our measure is the same as that of Pierce et al.^2^. Pearson correlation coefficients between the first question and the second, and third question within this sample were 0.68490 (p<0.0001) and 0.67067 (p<0.0001). These findings support the validity of our measure.

#### Independent variables

Sociodemographic measures included age, sex, ethnicity, father’s and mother’s occupation, type of university, and monthly expenses. University type was determined using the China university ranking system (‘Higher level,’ ‘Middle level,’ or ‘Lower level’) as set out by the National Ministry of Education^25^. A monthly expense (RMB, China currency) was measured through a question: ‘How much money do you spend each month?’.

Environmental factors were measured in two ways. The first was exposure to secondhand smoke (SHS) in private (domestic) and public places, while the second focused on cigarette advertisement and cigarette promotion activities. Students were asked whether they had been exposed to SHS in the past week either at home or in public places and whether they had seen cigarette promotional activities or advertisements on campus in the past six months (Table 1).

**Table 1:** Sociodemographic characteristics of sample, the prevalence of susceptibility to smoking, and unadjusted odds ratio (OR)

*Group*	*N*	*Percent of sample*	*Prevalence (%)*	*Unadjusted OR ( 95% CI)*
**Sociodemographic variables**				
Age (years)				
<20	1605	11.0	22.4	1.00
20-	1890	17.8	19.2	0.85 (0.61–1.17)
21-	2208	24.7	21.9	0.97 (0.72–1.30)
22-	2012	22.1	23.5	1.06 (0.79–1.43)
23-	2057	24.6	26.6	1.45 (1.02–2.61)*
Gender				
Male	2959	31.0	41.9	1.00
Female	6858	69.0	14.0	0.24 (0.20–0.28)**
Father’s occupation				
Worker, farmer, and business	7790	79.2	23.6	1.00
Manager and staff	1406	14.7	20.4	0.73 (0.56–0.93)*
Teacher and science and technical worker	621	6.0	21.9	1.10 (0.83–1.45)
Mother’s occupation				
Worker, farmer, and business	7910	80.1	23.4	1.00
Manager and staff	1253	12.7	21.5	0.90 (0.71–1.13)
Teacher and science and technical worker	654	6.8	21.4	0.89 (0.68–1.17)
Ethnicity				
Han	9214	95.3	23.1	1.00
Minority	603	4.7	21.4	0.90 (0.66–1.22)
Monthly expenditure (RMB)				
<500	1152	9.3	22.4	1.00
500–999	5152	54.2	22.7	1.02 (0.70–1.48)
1000–1499	2815	29.5	23.7	1.08 (0.72–1.62)
≥1599	718	6.9	23.4	1.06 (0.68–1.67)
University type				
Higher level	3597	43.1	21.0	1.00
Middle level	5683	55.5	24.8	1.24 (0.92–1.698)
Lower level	537	1.5	19.0	0.89 (0.70–1.12)
**Exposure to SHS**				
Exposure in domestic places in past week				
No	7338	83.4	12.6	1.00
Yes	1578	16.6	26.4	1.94 (1.63–3.31)**
Exposure in public places in past week				
No	4805	55.4	14.0	1.00
Yes	4111	44.6	16.0	1.16 (1.02–1.31)*
**Cigarette advertisement and promotion**				
Did you see cigarette promotional activities on campus in the past 6 months?				
No	9212	95.1	22.3	1.00
Yes	461	4.9	37.1	2.06 (1.42–2.94)**
**Cigarette advertisement and promotion**				
Did you see cigarette advertisements on campus in past 6 months?				
No	9104	94.1	22.0	1.00
Yes	570	5.9	38.1	2.42 (1.57–3.55)**
**Attitude toward health professions (HPs) tobacco control**				
Should HPs obtain specific training on cessation techniques?				
No	237	7.5	38.4	1.00
Yes	9080	92.5	21.8	0.45 (0.37–0.55)**
Should HPs serve as role models for their patients and the public?				
No	819	8.8	33.8	1.00
Yes	8998	91.2	22.0	0.55 (0.45–0.57)**
Should HPs routinely advise patients to stop smoking?				
No	442	4.2	38.9	1.00
Yes	9375	21.4	22.4	0.45 (0.33–0.63)**
Should HPs advise patients who use other tobacco products to quit using these products?				
No	5362	54.4	21.6	1.00
Yes	4455	45.6	24.8	1.20 (1.07–1.35)*
Do HPs have role in providing advice or information about smoking cessation to patients?				
No	1257	12.3	28.8	1.00
Yes	8560	87.7	22.2	0.71 (0.57–0.88)**
Are patient chances of quitting smoking increased with advice from HP?				
No	1213	12.0	22.3	1.00
Yes	8604	88.0	22.3	0.72 (0.58–0.89)**
**Curriculum and practices**				
In school training, did you receive information about the dangers of smoking?				
No	1802	17.1	27.5	1.00
Yes	8015	82.8	22.1	0.75 (0.60–0.93)**
Have you ever heard of nicotine replacement therapies in tobacco cessation programs?				
No	5412	52.1	23.9	1.00
Yes	4405	47.9	23.1	0.98 (0.83–1.17)
Light cigarettes are less harmful to health				
Do not agree	6077	66.3	13.1	1.00
Agree	2839	33.7	18.5	1.51 (1.22–1.89)**
Do you care about others smoking around you?				
No	445	4.4	47.4	1.00
Care	4311	44.3	26.5	0.61 (0.50–0.74)**
Very	5061	51.3	18.0	0.24 (0.17–0.36)**
Did you advise your family members to stop smoking?				
No	3131	47.3	25.8	1.00
Yes	3687	52.7	19.5	0.70 (0.58–0.84)**

* p<0.05

** p<0.01.

Students attitudes towards the role of health professionals in tobacco control were measured using six questions relating to whether physicians should obtain specific training on cessation techniques, serve as role models for their patients and the general public, give advice to their patients to stop smoking and whether they thought that such advice would be effective in helping patients quit (Table 1). In addition, five questions were also asked about the nature of their curriculum and their subsequent cessation interventions with respect to family members. The influence of the curriculum was assessed by asking about receipt of knowledge about the dangers of smoking and knowledge of the harmful health effects of light cigarettes. Respondents’ behaviour related to tobacco was assessed with questions on whether they cared about SHS exposure and advised family members to stop smoking.

### Data analysis

All data were entered into a database using Microsoft Excel. The data were then imported into SAS (9.3 version) for statistical analyses. Descriptive statistics were calculated to determine the prevalence of susceptibility to smoking. A logistic model was used to assess associations between the dependent and independent variables. Both unadjusted and adjusted methods were considered in the data analyses. The unadjusted method used only the key factors of interest as independent variables in the analyses, while the adjusted method added all of the possible confounders listed in Table 1 as covariates in the logistic models. We used binary logistic regression estimated with the maximum likelihood function. We present the final model with all covariates regressed on the dependent variable because we were cautious that step-wise inclusion (or its reverse) may be suspicious of p-hacking and the arbitrary discard of undesirable variables, which has elicited much controversy in current studies^26^. We also added OR and 95% CI in the statistic analysis section.

SAS survey logistic procedures were applied in the above analyses, using the university as the clustering unit, in order to account for a within-clustering correlation, attributable to the complex sample for unadjusted analysis. Series models were built for each primary predictor, with adjustment for the influence of potentially confounding sociodemographic characteristics in multiple variables logistic regression.

All analyses were weighted. Weights included: 1) sampling weights, as the inverse of the probability of selection, calculated at university; and 2) post-stratification weights, calculated in relation to sex, based on estimated distributions of this characteristic from a national survey^27^. The final overall weights were computed as the product of the above two weights.

## RESULTS

Valid questionnaires were completed by 97.6% of the potential students, resulting in a sample of 11954 students from 50 different universities. Subsequent analysis focused on 8916 non-smokers among medical students. Eleven percent of the resulting sample was less than 20 years of age, 19%, 22%, and 25% were either 20, 21 and 22 years old, with the remainder of participants being more than 23 years old. Of the study sample, 31% were male, and 69% were female. In total, 95% of the participants were Han Chinese (Table 1).

The prevalence of susceptibility to smoking was 23.0% (95% CI: 20.4–25.8%). The unadjusted logistic analysis showed: those who were older, male, and whose fathers’ occupations were workers, farmers, and in business were more likely to be susceptible to smoking. Exposure to SHS in living and public places, cigarette advertisement, and promotional activities on campus were positively associated with susceptibility to smoking. All attitudes toward health professionals involved in tobacco control were negatively associated with susceptibility to smoking. For example, students who thought that health professionals should serve as role models or obtain specific training in cessation techniques were much less likely to be susceptible to smoking, the respective ORs were 0.55 (95% CI: 0.45–0.57) and 0.45 (95% CI: 0.37–0.55). For the curriculum and behavioural variables, all except ‘nicotine replacement therapies’ were associated with susceptibility to smoking (Table 1).

Multilevel logistic regression confirmed that exposure to SHS in domestic and public places and the level of cigarette advertisements/promotional activities on campus were positively associated with susceptibility to smoking. All variables related to attitudes, except ‘advising patients who use other tobacco products’, were associated with susceptibility to smoking. Those who received information about the dangers of smoking, and did not agree that light cigarettes are less harmful to health were less susceptible to smoking. Caring about the effects of SHS and advising family members to stop smoking were negatively associated with susceptibility to smoking (Table 2).

**Table 2:** Results of multiple logistic regression

*Group*	*N*	*Prevalence (%)*	*Adjusted OR ( 95% CI)*
**Exposure to SHS**			
Exposure in domestic places in past week			
No	7338	12.6	1.00
Yes	1578	26.4	1.63 (1.20–2.20)**
Exposure in public places in past week			
No	4805	14.0	1.00
Yes	4111	16.0	1.78 (1.08–2.93)*
**Cigarette advertisement and promotion**			
Did you see cigarette promotional activities on campus in past 6 months?			
No	9212	22.3	1.00
Yes	461	37.1	1.90 (1.30–2.78)**
Did you see cigarette advertisement on campus in past 6 months?			
No	9104	22.0	1.00
Yes	570	38.1	1.91 (1.33–2.75)**
**Attitude toward health professions (HPs) tobacco control**			
Should HPs obtain specific training on cessation techniques?			
No	237	38.4	1.00
Yes	9080	21.8	0.52 (0.42–0.65)*
Should HPs serve as role models for their patients and the public?			
No	819	33.8	1.00
Yes	8998	22.0	0.61 (0.48–0.80)**
Should HPs routinely advise patients to stop smoking?			
No	442	38.9	1.00
Yes	9375	22.4	0.49 (0.35–0.70)**
Do HP have role in providing advice or information about smoking cessation to patients?			
No	1257	28.8	1.00
Yes	8560	22.2	0.73 (0.57–0.94)*
Are patient chances of quitting smoking increased with advice from HP?			
No	1213	22.3	1.00
Yes	8604	22.3	0.78 (0.63–0.97)*
**Curriculum and behaviors**			
In school training, did you receive information about the dangers of smoking?			
No	1802	27.5	1.00
Yes	8015	22.1	0.75 (0.60–0.95)*
Light cigarettes are less harmful to health			
Do not agree	6077	13.1	1.00
Agree	2839	18.5	1.27 (1.07–1.64)**
Do you care about others smoking around you?			
No	445	47.4	1.00
Care	4311	26.5	0.68 (0.56–0.84)**
Very	5061	18.0	0.33 (0.23–0.49)**
Did you advise your family members to stop smoking?			
No	3131	25.8	1.00
Yes	3687	19.5	0.81 (0.68–0.97)*

* p<0.05

** p<0.01.

## DISCUSSION

To our knowledge, this is the first study examining susceptibility to smoking and determinants among medical students in China and elsewhere in the world. In this study, we estimated the susceptibility to smoking among never-smoking medical students and also identified associated factors.

The first aim of the study addressed the question of quantifying the prevalence of susceptibility to smoking among medical students. This study found that the prevalence of susceptibility to smoking among never-smoking medical students was 23.0%. This prevalence was higher than the prevalence (12.5%) in adolescents (Global Youth Tobacco Survey)^2,28^. This high prevalence of susceptibility may in part explain the high smoking prevalence observed among practising physicians in China. Though the higher smoking prevalence among physicians in China may be associated with social factors^16^, their attitudes and practices of smoking gradually form and become inseparable from their early experiences^22^. This high prevalence of susceptibility is problematic, especially if susceptibility leads to subsequent initiation and the continuation of a relatively high rate of smoking among Chinese medical students. We intend to study how future medical professionals are susceptible to smoking; we believe this association can be built because the pipeline from medical students to medical professionals is very saturated and very few medical students work outside the healthcare industry including public hospitals, private clinics, and other healthcare units^24^. While not all medical students eventually settle into employment as physicians, all physicians were previous medical students. Susceptibility to smoking among medical students ultimately shapes the initial experience about the normativity of smoking for the vast majority of healthcare workers. Many studies have shown that susceptible adolescents were two to three times more likely to experiment with cigarettes later on than non-susceptible adolescents^2,5,6^. This should also be true for medical students. Thus, it is important to implement early intervention efforts and prevent never-smoking medical students from initiating smoking and transitioning to regular smokers.

Our finding that older males in particular had a higher risk of susceptibility to smoking is consistent with normative patterns in China where smoking is an accepted adult practice predominantly among males compared to females^29^. Given that being susceptible to smoking is an important risk factor for future smoking behaviour, targeted smoking prevention efforts during medical training are warranted. Based on our study results, targeted efforts directed toward older male students should be a priority.

The second important aim of the study was to examine the role of environmental factors in susceptibility to smoking. The findings showed that exposure to SHS at home and in public places was significantly associated with increased susceptibility to smoking among never-smoking individuals. This finding is consistent with studies from other populations where smoking in public and private spaces helps reinforce pro-smoking norms^2,3^. Not surprisingly, we also found that those who care about SHS were less likely to be susceptible to smoking. Consistent with this result, Mayhew et al.^30^ reported that experiencing physical and/or unpleasant reactions to SHS exposure predicted lower risk for smoking susceptibility^30^. Exposure to SHS causes many serious diseases. However, millions of non-smokers remain exposed to SHS in their homes, workplaces, public places, and vehicles. The World Health Organization (WHO) has estimated that in 2004, about one-third of adults and 40% of children worldwide were exposed to SHS^31^.

Smoke-free policies are the most effective way to reduce exposure to tobacco smoke among the public^31^. Given that most medical students live on campus, study findings support the need to create smoke-free campuses, to protect young adult never-smokers from exposure to SHS. In medical universities, a smoke-free campus policy would also promote a healthy campus environment for students, faculty, staff, and visitors, support and encourage cessation attempts among smokers trying to quit, and indicate strong support for tobacco control among university leaders^22^. In addition, a smoke-free campus is a good way to strengthen attitudes toward protecting others from SHS and support medical student involvement in promoting tobacco control. Medical students could be encouraged to advocate a smoke-free campus policy and participate in related activities. The study findings also support the need to implement comprehensive tobacco bans along with stronger regulations of tobacco company advertising practices. The FCTC requires that each party to the Convention ‘undertake a comprehensive ban, and restrict tobacco advertising, promotion, and sponsorship on radio, television, print media, and also, as appropriate, other media such as the Internet’^17^. Several studies have documented that complete bans on tobacco advertising and promotion can protect youth from initiating smoking and adults from continuing to smoke^32,33^. Many studies have also found clear associations between cigarette advertisement/promotion and susceptibility to smoking among adolescents^34-36^. This study provided evidence for this relationship. With knowledge of the health risks of smoking and tobacco control measures, medical students could be involved in lobbying against cigarette advertisement/promotion, and restricting the sale of tobacco products on campuses as part of nation-wide efforts to introduce policies to ban tobacco advertising/promotion activities in public places in China.

For our third aim, this study tested students’ tobacco control attitudes, curriculum influences, and other behaviours associated with susceptibility to smoking. We found that the positive attitudes toward health professionals’ role in tobacco control included in this study were commonly associated with low susceptibility to smoking. Susceptible, never smokers held stronger negative opinions about health professionals’ role in tobacco control than never smokers who were not susceptible to smoking. Previous research has demonstrated that anti-smoking norms and attitudes are related to lower smoking prevalence^37,38^. Furthermore, receipt of information about the dangers of smoking was negatively associated with susceptibility to smoking while thinking that ‘light cigarettes are less harmful to health’ was positively associated with susceptibility to smoking. Together, these study findings underscore the importance of strengthening medical education related to the role of physicians in tobacco control as well as knowledge and skills related to supporting cessation. It is likely important to include this circular content early in medical programs before students progress to becoming current smokers. Such training is important because it not only influences students’ susceptibility to smoking but also may enhance awareness of physician roles in tobacco control, as well as attitudes and behaviours regarding smoking cessation.

Medical professions have the potential to play a critical role in tobacco control in relation to improving services, advocating policies and serving as role models for social change. Currently, there are approximately 190 universities offering programs in public health, nursing, and clinic medicine in China. Nearly 100000 students graduate annually from these faculties, with a significant number being employed by medical institutes^38^.

The tobacco epidemic is a major public health threat in China. Although the Chinese National People’s Congress ratified the Framework Convention on Tobacco Control (FCTC) in 2005, implementation of the FCTC recommended activities is progressing very slowly. This slow progress is related to the lack of skilled personnel who can act as advocators to promote anti-tobacco activities. This situation may be related to tobacco control training in medical schools. However, it is gratifying to see that this situation is beginning to change. As a result of this survey, ‘building advocacy capacity’ BI (the Bloomberg Global Initiative Project) projects have involved medical students in 98 universities in China. For example, a project focused on tobacco dependence treatment is in its last year. In this project, a general clinic smoking cessation program was development, and implemented in 10 hospitals, and embedded in the medical curriculum in 50 Universities. In these projects focusing on enhancing tobacco control and smoking cessation capacity training, smoke-free campuses are being implemented. These projects have effectively increased the capacity of the medical community in tobacco control advocacy^38,39^, and contributed to China’s tobacco control.

### Limitations

The study has strengths and limitations. It is the first study to estimate and identify factors associated with smoking susceptibility among never-smoking young adults on a national scale. The cross-sectional study design is an important limitation of our study. Therefore, a causal link between study variables and smoking susceptibility cannot be established. However, we employed a large sample, and our findings met several criteria for inferring causality, including the strength of some associations, their consistency, and plausibility of effect. Future studies need to compile longitudinal surveillance data to examine smoking susceptibility and initiation. A second study limitation is bias in our study, as some possible potential confounding factors were not controlled.

## CONCLUSIONS

This study provides new information about susceptibility to smoking and its determinants among medical students in China. This may help to partly understand the high smoking prevalence among physicians in China. These findings underscore the importance of conducting tobacco control training and establishing smoke-free campuses for reducing susceptibility to smoking among medical students.
